# Smoking behavior and nicotine dependence: the predictive role of personality traits

**DOI:** 10.3389/fpubh.2026.1860600

**Published:** 2026-07-20

**Authors:** Jaber S. Alqahtani

**Affiliations:** Department of Respiratory Care, Prince Sultan Military College of Health Sciences, Dammam, Saudi Arabia

**Keywords:** nicotine dependence (ND), personality traits, Saudi Arabia, smoking—epidemiology, smoking behavior, COPD, respiratory therapy

## Abstract

**Background:**

Smoking behavior and nicotine dependence remain significant global public health problems. Yet, variations in individual vulnerability cannot be entirely explained by sociodemographic factors alone. Personality traits may influence an individual's smoking behavior and nicotine dependence. Thus, this research investigates how fundamental personality traits impact smoking habits and nicotine addiction in adults, aiming to identify trait profiles linked to increased risk.

**Methods:**

This study was a cross-sectional survey targeting individuals with a smoking history, exclusively regular cigarette users. It used the validated Fagerström Test for Nicotine Dependence (FTND) to evaluate nicotine dependence, and the Big Five Inventory−10 (BFI-10) to assess personality traits. Multivariate regression models were used to assess associations.

**Results:**

556 smokers were included, with around half of the participants (46.8%) aged 18–30; (85%) reported no comorbidities, and 44% had completed secondary school. The smoking history was 20 ± 22 pack-years, while the FTND score was 5 ± 2.37, indicating a moderate level of dependence. Only two personality traits were significantly associated with the nicotine dependence score: conscientiousness and neuroticism. With each 1-unit increase in conscientiousness score, nicotine dependence decreases by 0.251 points (*p* = 0.011), highlighting a protective association. In addition, each one-unit increase in neuroticism score corresponds to a 0.233 unit increase in nicotine dependence (*p* = 0.018), demonstrating a strong positive correlation. Further, age and educational degree were negatively associated with nicotine dependence. Ex-smokers scored 0.34 points significantly higher than current smokers on conscientiousness (3.36 ± 0.83 vs. 3.02 ± 0.93, *p* value =0.005), a trait linked to health-promoting behaviors such as smoking cessation. There was a negative correlation between agreeableness score and pack-years (*r* = −0.11, *p* = 0.005).

**Conclusion:**

Personality traits, specifically conscientiousness and neuroticism, were associated with nicotine dependence. Former smokers demonstrated significantly higher conscientiousness scores than current smokers. The relationship between low agreeableness and increased lifetime tobacco exposure may indicate that prosocial traits might limit long-term smoking habits. Recognizing high-risk personality profiles could improve targeted prevention strategies, aid in personalized cessation interventions, and add depth to behavioral models of tobacco use.

## Introduction

Each year, over 8 million people globally are killed due to smoking, based on the World Health Organization (WHO) estimations (1). Tobacco use is one of the main risk factors for disability globally, accounting for over 200 million disability adjusted life years in 2019 (2). While the prevalence of smoking has decreased in many high-income nations, the trends in cigarette consumption and nicotine addiction are still increasing in developing nations and are associated with high tobacco-related illness and death (3). This underscores the critical need for targeted prevention and cessation efforts that extend beyond conventional demographic and environmental risk elements (4). Boredom proneness and affective temperaments are among the psychological factors known to impede smoking cessation, highlighting the importance of considering individual psychological profiles in tobacco-control efforts (5). In the context of the Eastern Mediterranean Region, Saudi Arabia stands out as a significant area of concern, given its high prevalence of smoking among the population and its associated health burden (6–11).

An increasing amount of research suggests that personality traits are significant psychological factors associated with the onset, continuation, and quitting of smoking (12). A longitudinal analysis revealed that smokers exhibited higher scores in neuroticism and extraversion compared to non-smokers (13). A further meta-analysis indicated that smokers exhibited higher levels of neuroticism and extraversion, while showing lower levels of conscientiousness compared to non-smokers (12). Additionally, a greater likelihood of relapse to smoking was linked to elevated neuroticism (12). The health behavior model of personality stands out as a prominent theory that explores how personality influences an individual's health outcomes (14). This model suggests that specific personality traits, especially conscientiousness and neuroticism, are linked to behaviors that can either enhance or harm health, such as drinking, ultimately influencing health outcomes (15–17). Therefore, the connection between personality and mortality and morbidity is mediated, essentially, by health behaviors (18). The Big Five personality traits (Openness, Conscientiousness, Extraversion, Agreeableness, Neuroticism) are associated with smokers' behavior and attitudes, with each trait impacting nicotine use in distinct ways (19). Nevertheless, one study indicates that these correlations may differ in direction and intensity across different ethnic and sociodemographic groups (20). This potentially indicates that cultural factors alter the extent to which personality impacts smoking habit and dependency.

In Saudi Arabia, no studies have investigated how personality traits predict smoking behavior and nicotine dependence, even though international studies increasingly highlight personality as a key factor in smoking use (21, 22). The unique cultural, social, and environmental factors of the Saudi Arabian population could influence the connection between personality traits and tobacco use in ways that studies from other regions may not fully address. Therefore, this study seeks to investigate the association between the Big Five personality traits, smoking behavior, and nicotine dependence among smokers in Saudi Arabia. The aim is to produce evidence that can guide culturally tailored cessation interventions and inform public health policy within the region.

## Methods

A cross-sectional study utilizing an electronic self-administered questionnaire was conducted to collect data. This study included people with a smoking history from the Kingdom of Saudi Arabia, exclusively regular cigarette users. The inclusion criteria were smokers aged ≥18 years. The IRB committee at Prince Sultan Military College of Health Sciences approved the study (IRB-2024-RC-026). It also adhered to the ethical guidelines of the Declaration of Helsinki.

### Survey tool and data collection

The study used a structured online questionnaire to gather data. The survey was available in English and Arabic, hosted on Google Forms. The survey link reached the broadest possible audience through social media and professional networking sites. All participants provided informed consent before completing the survey electronically. The participants received information regarding the study's objectives, the anticipated duration of the survey, data protection measures, and their right to participate voluntarily. The participant eligibility was verified through self-report screening items at the start of the questionnaire. Duplicate responses were prevented by restricting one response per device, while incomplete questionnaires, if available, were excluded prior to analysis

The survey has three parts: demographic information, the Fagerström Test for Nicotine Dependence (FTND) to evaluate nicotine dependence, and the Big Five Inventory−10 (BFI-10) to assess personality traits.

The study collected demographic details on age, gender, region, educational background, health status, and smoking history. In the second part, the study used the FTND as the assessment tool for nicotine dependence, using its validated six-item scale (23). The FTND assesses both behavioral and physiological aspects of nicotine use. The FTND scoring system ranges from 0 to 10 points, with higher scores indicating greater nicotine dependence (23). The FTND uses the following dependence categories to evaluate scores: very low (0–2 points), low (3–4 points), moderate (5 points), high (6–7 points), and very high (8–10 points). The FTND shows reliable results and valid measurements across various study groups (23). Regarding personality traits, the BFI-10 was used to measure them, a validated tool commonly used in the literature (24). The BFI-10 scale contains 10 items that measure the five personality dimensions through two items per trait (Extraversion, Agreeableness, Conscientiousness, Neuroticism, and Openness to Experience). The Likert scale used for rating items extends from 1 “strongly disagree” to 5 “strongly agree.” The two items for each trait are averaged to obtain the trait score out of 5. The BFI-10 provides reliable results and valid outcomes in research studies while remaining appropriate for large surveys that need to reduce participant burden (24). Reliability (Cronbach's α) indices were not computed for the BFI-10 subscales in line with the developers' explicit guidance, as it could lead to underestimate the true reliability (24). This is because each domain is measured by only two items deliberately selected to capture distinct content facets of the trait rather than to maximize inter-item homogeneity. Regarding the Arabic version, we adapted it from Research Group (25). International Social Survey Programme: work Orientation III—ISSP 2005, which followed the systematic translation and harmonization procedures used for international comparative surveys (25). A dedicated Arabic psychometric validation for BFI-10 was not applied as such validation falls beyond the scope of the present study.

### Power calculation?

According to the most recent data, there are around 5 million smokers in Saudi Arabia (26). Therefore, the study's minimum sample size will be 388 participants, assuming a 50% response distribution, a 5% margin of error, and a 95% confidence level.

### Statistical analysis

The researcher analyzed the data through IBM SPSS Statistics version 28.0 (IBM Corp., Armonk, NY, USA). The study used descriptive statistics to present participant information for demographic characteristics, smoking history, nicotine dependence levels, and personality trait scores. Spearman's correlation was used to assess the correlation between nicotine dependence score, smoking history, and mean scores of personality traits. An independent-samples *t*-test was used to compare mean scores for different personality traits between smokers and former smokers, with Bonferroni adjustment for multiple comparisons. The analysis used multiple linear regression to identify predictors of nicotine dependence, treating the FTND total score as the dependent variable and the five personality traits as independent variables. The model was adjusted for age, gender, education, smoking status, and history. Assessment of residual assumptions, and multicollinearity diagnostics were applied to evaluate the adequacy of the regression model. The statistical significance threshold for all analyses was set at *p* < 0.05.

## Results

A total of 556 smokers were included; most were young adults, with around half (46.8%) aged 18–30. By region, the sample was distributed across Saudi Arabia, with the western region contributing the largest share (41%), followed by the eastern region (27%) (Table 1). Most participants reported having no chronic medical conditions, and 44% had completed secondary school. Around 83% of participants were current smokers, and the rest were former smokers. The smoking history was 20 pack-years, with a mean nicotine dependence score of 5, suggesting moderate dependence overall. Table 2 summarizes the mean scores of all assessed personality traits.

**Table 1:** Sample characteristics (*n* = 556).

Demographic data	*N* (%)or mean ±SD
Gender	
Male	495 (89%)
Female	61 (11%)
What is your age range (year)	
18–30	260 (46.8%)
31–40	114 (20.5%)
41–50	103 (18.5%)
>50	79 (14.2%)
Region	
Eastern	153 (27%)
Western	226 (41%)
Central	73 (13%)
Northern	66 (12%)
Southern	38 (7%)
Comorbidities	
Cancer	3 (0.5%)
Chronic respiratory disease	12 (2%)
Diabetes mellitus	15 (3%)
Heart disease	8 (2%)
Hypertension	32 (6%)
Depression	7 (1%)
Renal disease	7 (1%)
No chronic diseases	472 (85%)
Highest level of formal education	
Secondary school	242 (44%)
Diploma	111 (20%)
Bachelor's	185 (33%)
Post graduate studies	18 (3%)
Smoking status	
Current smoker	463 (83%)
Former smoker	93 (17%)
Pack-years	20 ± 22
Nicotine dependence score	5 ± 2.37

**Table 2:** Personality background of the sample based on the Big Five Inventory (BFI) scale (*n* = 556).

Personality characteristics	Mean ±SD
big five inventory (1–5 scale)	
Openness	3.14 ± 0.83
Conscientiousness	3.07 ± 0.92
Extraversion	3.03 ± 0.76
Agreeableness	3.22 ± 0.89
Neuroticism	3.03 ± 0.90

### Correlation between nicotine dependence score and personality characteristics

The study used Spearman's rho correlations to study how nicotine dependence relates to personality characteristics. The finding showed that nicotine dependence scores had a negative relationship with conscientiousness (ρ = −0.11, *p* = 0.008) and a positive relationship with neuroticism (*r* = 0.14, *p* < .001). The analysis showed no meaningful relationships between nicotine dependence and extraversion (*r* = −0.01, *p* = 0.75), agreeableness (*r* = 0.07, *p* = 0.08), or openness to experience (*r* = −0.05, *p* = 0.16).

### Correlation between smoking history and personality characteristics

The study established a negative relationship between agreeableness score and smoking history, as measured by pack-years (*r* = −0.11, *p* = 0.005). The analysis revealed no substantial relationships between pack-years and extraversion (*r* = 0.009, *p* = 0.83), conscientiousness (*r* = 0.03, *p* = 0.48), neuroticism (*r* = 0.05, *p* = 0.17), and openness to experience (*r* = −0.02, *p* = 0.63).

### Correlation between nicotine dependence score and smoking history

The analysis showed that total nicotine dependence scores positively correlated with pack-years consumption (*r* = 0.393, *p* < 0.001). Hence, people with higher nicotine dependence levels smoked more cigarettes during their lifetime.

### Comparison of big five personality trait mean scores between current and former smokers

**Figure 1:**
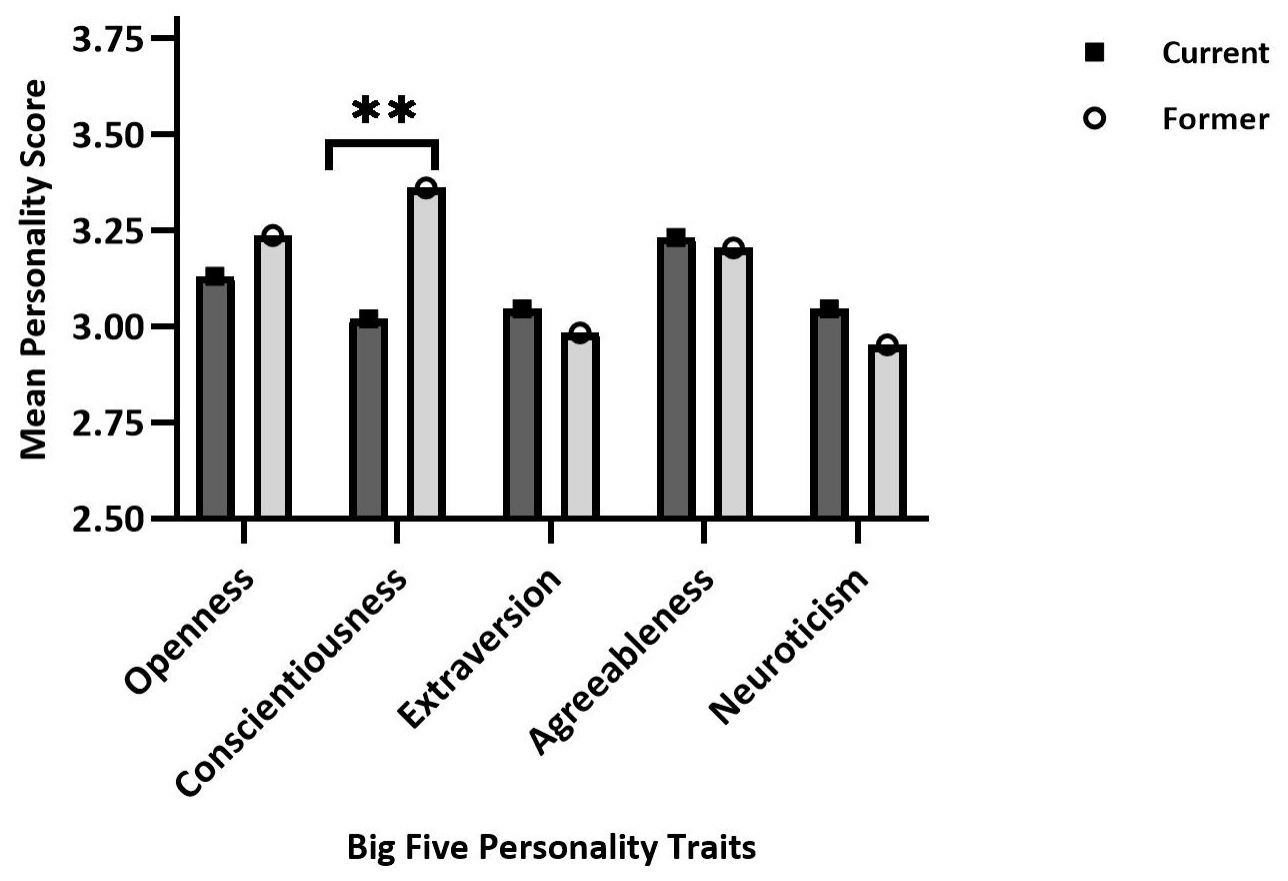
Difference in mean scores across the big five personality traits between former and current smokers. ** indicated *p* value = 0.005.

When comparing former and current smokers on mean personality trait scores, a statistically significant difference was only observed in conscientiousness, with ex-smokers scoring approximately 0.34 points higher than current smokers (3.36 ± 0.83 vs. 3.02 ± 0.93, *p* value post Bonferroni correction (*p* = 0.005). No significant differences were observed in the other traits, as indicated by *p*-values >0.05. Figure 1 shows the difference in mean scores across the Big Five personality traits between former and current smokers.

### Association between nicotine dependence score and personality characteristics

A multiple linear regression was applied to study how personality characteristics relate to nicotine dependence [full model-fit indices (*R* = 0.495, *R*^2^ = 0.245, adjusted *R*^2^ = 0.231, SEE = 2.078)]. Tolerance values ranged from 0.792 to 0.989, and VIF values ranged from 1.01 to 1.26, all well within acceptable thresholds (Table 3). These results indicate that there is no problematic multicollinearity among the predictors. Residual diagnostics showed no violations of assumptions. Standardized residuals ranged from −3.01 to 2.47 with a mean near zero, indicating no influential outliers and supporting normality and homoscedasticity. Predicted values were reasonable, and residuals were centered around zero, confirming unbiased model predictions.

**Table 3:** Linear regression output for exploring the impact of personality dimensions on nicotine dependence score, after adjusting for sociodemographic and lifestyle variables.

Variables	*B*	SE	95% CI	*P*-value	Tolerance	VIF
Openness	−0.053	0.10	−0.26 to 0.15	0.622	0.97	1.03
**Conscientiousness**	**−0.251**	**0.09**	**−0.44 to** **−0.05**	**0.011**	0.94	1.05
Extraversion	−0.008	0.11	−0.23 to 0.22	0.946	0.97	1.02
Agreeableness	0.167	0.10	−0.03 to 0.37	0.107	0.90	1.10
**Neuroticism**	**0.233**	**0.09**	**0.04 to 0.42**	**0.018**	0.97	1.02
**Age**	**−0.290**	**0.09**	**−0.46 to** **−0.11**	**0.001**	0.79	1.26
Gender	0.133	0.28	−0.42 to 0.69	0.640	0.98	1.01
**Pack-years**	**0.047**	**0.01**	**0.03 to 0.05**	**<0.001**	0.85	1.16
**Smoking status**	**0.680**	**0.24**	**0.20 to 1.15**	**0.005**	0.96	1.04
**Education level**	**−0.370**	**0.09**	**−0.56 to** **−0.18**	**<0.001**	0.92	1.08

SE, Standard error; VIF (Variance Inflation Factor). In the smoking status, former smokers were used as reference value.

There are only two personality traits that were associated with the nicotine dependence score: conscientiousness and neuroticism. For each single-point increase in conscientiousness score, there is a 0.251-point reduction in nicotine dependence (*p* = 0.011), indicating a protective relationship. In addition, each one-unit increase in neuroticism score corresponds to a 0.233 unit increase in overall nicotine dependence (*p* = 0.018), demonstrating a strong positive correlation. On the other hand, the regression showed that extraversion, agreeableness, and openness to experience did not predict nicotine dependence. Table 3 shows other significant predictors for nicotine dependence, including a negative association with age and educational degree, and a positive association with pack-years and current smokers.

## Discussion

For the first time in Saudi Arabia, this study explored the association between the Big Five personality traits and nicotine dependence in smokers. The findings show that nicotine dependence was significantly associated with only two personality traits, namely conscientiousness and neuroticism, within the regression model. A higher level of conscientiousness was linked with former smokers. Additionally, only the agreeableness trait was significantly correlated with smoking history, measured as pack-years. The findings align with the existing international literature that links personality traits to smoking behaviors and dependence.

The study demonstrated a strong negative connection between conscientiousness and FTND scores (ρ = −0.11, *p* = 0.008). Regression analysis confirmed that each unit increase in conscientiousness resulted in a 0.222-point drop in nicotine dependency. This protective function of conscientiousness is among the most frequently reported findings in the personality and smoking literature. This aligns with a 10-year longitudinal study that reported that the conscientiousness trait reduces lifetime cigarette use, progression to daily smoking, and smoking persistence, demonstrating that it protects against both progression and maintenance (27). A study of Chinese smokers also found a negative association between the conscientiousness trait and smoking behavior, specifically quitting (28). Another longitudinal study conducted in the Czech Republic found that individuals with elevated levels of conscientiousness during childhood were less likely to smoke regularly in young adulthood (29). The finding was further confirmed by another study conducted among Lebanese student smokers, which found that more conscientious smokers are less likely to have high cigarette dependence (30). Moreover, a meta-analysis revealed that individuals who have smoked in the past, as well as those who currently smoke, exhibit lower levels of conscientiousness compared to individuals who have never smoked (12). Overall, previous work from different countries suggests that those with greater levels of conscientiousness are less likely to practice health-risk activities like smoking. Such findings support our finding that Ex-smokers scored 0.34 points significantly higher than current smokers on conscientiousness (3.36 ± 0.83 vs. 3.02 ± 0.93, *p* value <0.001), a trait linked to health-promoting behaviors such as smoking cessation. This highlights the importance of the conscientiousness trait in smoking prevention strategies.

Another main finding of this study is that the neuroticism trait was positively associated with nicotine dependence. This aligns with substantial evidence indicating that neuroticism is among the strongest personality-level predictors of nicotine dependence. A meta-analysis involving more than 79,000 subjects revealed a significant association between higher neuroticism and current smoking as well as smoking relapse among former smokers and reduced odds of cessation, highlighting its influence throughout the smoking trajectory (12). This association is fundamentally grounded in the models of addiction that emphasize negative reinforcement and tension reduction. People who exhibit high levels of neuroticism might turn to smoking to alleviate tension, utilizing nicotine as a way to cope with anxiety, irritability, and emotional instability (31). A comprehensive meta-analysis of 25 published studies reveals that smokers exhibit higher levels of neuroticism compared to non-smokers (32). Furthermore, prospective studies have indicated that high levels of neuroticism may be linked to future smoking (18, 31). A meta-analysis identified an association between neuroticism and difficulties in smoking cessation, as well as an increased likelihood of re-smoking among individuals who have previously quit (12). Nevertheless, the relationship between neuroticism and nicotine dependence may not be direct, but it is mediated by emotional disturbance, as evidenced by the fact that neuroticism primarily influences dependence through depressed symptoms (33). This holds significant relevance for the Saudi context, as stress emerged as the leading factor for smoking initiation among smokers in Saudi Arabia (34). This indicates that the role of smoking in regulating emotions may be especially significant within the Saudi population.

The current study revealed a significant negative correlation between agreeableness and pack-years. This suggests that smokers who exhibit lower levels of agreeableness tend to have a longer smoking history. A meta-analysis involving over 4,000 subjects indicated that low levels of agreeableness were linked to current smoking behaviors (32). Nevertheless, prior research has not established a connection with smoking history measured by pack-years. Given that agreeableness did not show a significant correlation with nicotine dependence in our study, yet was connected to smoking history, it may be more accurately related to the persistence and duration of smoking behavior rather than its intensity at any particular time. Additionally, a substantial longitudinal investigation involving 15,572 participants with a follow-up period from 4 to 20 years, in contrast to non-smokers, individuals who currently smoke exhibited a greater likelihood of experiencing a decline in agreeableness over time (35). This finding may imply a bidirectional relationship, wherein smoking may actively contribute to the further deterioration of prosocial characteristics throughout adulthood. Within the Saudi culture, where the importance of social unity and adherence to family and community standards is paramount, low agreeableness could serve as a key risk factor for non-stop smoking. This is because individuals who are less responsive to interpersonal disapproval might show greater resistance to the social pressures intended for quitting smoking. Thus, efforts to reduce non-stop smoking should also take into account the interpersonal and social-norm aspects associated with agreeableness.

It is noteworthy to mention that certain sociodemographic variables were associated with nicotine dependence. The study indicated that there were negative associations between age and nicotine dependence, suggesting that as age increases, dependence tends to decrease. Additionally, a higher educational degree has been associated with a reduction in nicotine dependence. These findings align with earlier research indicating that younger individuals and those with a lower level of education tend to smoke more heavily, show higher dependence scores, and experience less effective cessation attempts (36–38). The identified connections emphasize the need to implement a holistic management approach that may consider individual personality traits and associated sociodemographic factors.

These findings hold significant importance from a public health standpoint, especially considering the established impact of smoking in Saudi Arabia. A systematic review reports that between 2 and 40% of Saudi adults are current smokers, with a greater prevalence observed among men and younger individuals (8). Additionally, smoking initiation often takes place during adolescence and early adulthood and could lead to poor academic and health outcomes (39–41). Previous Saudi research found that age, gender, wealth, peer and family smoking, and marital status were important predictors of tobacco use, but personality characteristics were not used (8, 42). This study demonstrates that personality evaluation may enhance risk categorization beyond demographic and behavioral characteristics. Clinically, a short personality screening may assist in pinpointing smokers who are especially susceptible to dependence and who could gain from more focused or customized cessation support. This support might include cognitive-behavioral strategies for emotion regulation, stress management, planning, and self-monitoring.

This study has some limitations. The cross-sectional design does not allow for causal inferences on whether personality features increase nicotine dependency and pack-years exposure or not. The reliance on online convenience sampling may have resulted in self-selection bias. Factors related to smoking and personality were assessed via self-report, which may be biased by social desirability and recall. The sample was limited to Saudi smokers, making it culturally relevant but not applicable to other populations. The predominance of male participants in the sample limits the generalizability of the findings. Although several associations reached statistical significance, these effects are unlikely to be of major clinical magnitude at the individual level and should be interpreted with caution. Additionally, a formal Arabic psychometric validation for BFI-10 was not applied as such validation falls beyond the scope of the present study. Lack of formal psychometric validation could lead to culturally specific item-functioning effects that lead to measurement error. Thus, all personality findings are framed as exploratory, and prospective replication using a validated Arabic instrument is recommended. Use of brief personality instrument and possible residual confounding are limitations that should be acknowledged. Lastly, anxiety, depression, perceived stress, and concurrent substance use were not measured, and residual confounding from these unmeasured variables remains plausible. Despite the previous limitations, the study offers new evidence that conscientiousness and neuroticism relate to nicotine dependence, while agreeableness correlates with cumulative smoking exposure in Saudi smokers. To the author's understanding, there has not been a prior systematic examination in Saudi Arabia that assesses the Big Five personality traits in connection with nicotine dependence and pack-years.

Additionally, the findings are important because personality factors have often been neglected relative to sociodemographic and psychosocial predictors. Future research should investigate this with larger, more diverse samples and employ longitudinal designs. Incorporating personality-informed strategies into tobacco control plans can potentially improve the effectiveness of smoking cessation programs and help reduce tobacco-related diseases in Saudi Arabia.

## Conclusion

Personality traits, specifically conscientiousness and neuroticism, were associated with nicotine dependence, considering sociodemographic factors. Neuroticism showed a positive association with nicotine dependence, potentially reflecting difficulties in emotion regulation, whereas conscientiousness showed an inverse association. A greater level of conscientiousness was linked with former smokers. The relationship between low agreeableness and increased lifetime tobacco exposure suggests that prosocial traits might limit long-term smoking habits. These findings can potentially support the use of personality assessment in smoking cessation programs to create psychologically customized therapies that address smokers' trait-specific susceptibility. Future longitudinal research is required to verify the temporal and causal relationships between personality traits and nicotine dependence.

## Data Availability

The original contributions presented in the study are included in the article/supplementary material, further inquiries can be directed to the corresponding author.
